# Effects of voltage application on hydrogen production performance and stabilization in an electro-supported dynamic membrane bioreactor

**DOI:** 10.3389/fmicb.2026.1822655

**Published:** 2026-06-11

**Authors:** Young-Bo Sim, Sang-Hyoun Kim

**Affiliations:** 1 Department of Civil and Environmental Engineering, Yonsei University, Seoul, Republic of Korea; 2 Department of Civil and Environmental Engineering, Faculty of Engineering, Imperial College, London, United Kingdom

**Keywords:** biofilm, dark fermentation, electro-supported dynamic membrane bioreactor, hydrogen production, rapid stabilization time

## Abstract

**Introduction:**

A novel electro-supported dynamic membrane bioreactor (EDMBR) was by integrating electrode function into a dynamic membrane module made of a stainless steel mesh support with a pore size of 444 mm. This work aimed to investigate the effects of voltage application on stabilization time, hydrogen production performance, metabolic pathway shift, and microbial community change during continuous hydrogen production.

**Methods:**

Two independent EDMBR systems were operated under continuous conditions at different applied voltages ranging from 30 to 700 mV. Hydrogen production performance, soluble byproducts, suspended biomass, biofilm concentration, and microbial community were analyzed under different operational conditions.

**Results:**

Voltage application in the EDMBR influenced both process stabilization and biological responses during continuous biohydrogen production. Electrical input was associated with accelerated stabilization time through improved biomass retention, whereas sustained voltage application was related to a shift in metabolic pathways and changes in microbial community. Voltage application reduced the stabilization time required to reach pseudo-steady-state from 8 days to 3 days. However, excessive electrical input was associated with decreased hydrogen production performance and localized corrosion of the electro-supported mesh under elevated current density conditions.

**Discussion:**

These results indicate a trade-off between rapid stabilization and hydrogen production performance under voltage application, suggesting that electrical input should be strategically managed rather than continuously intensified. An operational window from 0.30 to 1.21 mA/cm^2^ was identified to balance rapid stabilization with hydrogen production performance while minimizing the risk of corrosion and reactor deterioration. Within this range, voltage application can be applied to restore pseudo-steady-state following reactor deterioration. However, excessive electrical input may increase the risk of metabolic pathway shifts and long-term performance decline. Therefore, further studies are recommended to develop operational strategies that maintain metabolic flux toward H2-producing pathways and preserve the dominance of H2-producing bacteria during continuous EDMBR operation.

## 1 Introduction

Hydrogen is considered a key alternative fuel due to its high energy density (122 kJ/g) and the absence of CO_2_ generation during combustion, which could significantly contribute to achieving global decarbonization and net-zero emission goals (Capurso et al., 2022; Hoang et al., 2023; Kouchaki-Penchah et al., 2024). In particular, green hydrogen technologies that satisfy carbon emission criteria during hydrogen production are attracting increasing attention, and significant progress has been achieved across various sectors. However, commercialization still faces challenges due to high dependence on renewable energy sources and insufficient economic feasibility (Dincer and Acar, 2014; Toledo-Alarcón et al., 2019).

Bioenergy production from organic waste is considered eco-friendly because CO_2_ emissions are offset by organic waste consumption during its life cycle (Calli et al., 2006). In particular, dark fermentation can directly produce hydrogen from organic waste without reforming, and it satisfies green hydrogen criteria based on life cycle analysis (LCA) from collection to purification (Lee et al., 2024). In this context, dark fermentation is considered a promising method for green hydrogen production due to its economic and practical advantages. Especially, continuous dark fermentative hydrogen production enables high hydrogen production rates, and reduces reactor volume and operational area for the same hydrogen production capacity (Kim et al., 2023; Sanghvi et al., 2024). However, deteriorated continuous systems often require prolonged stabilization or may even terminate operation, and this instability continues to hinder the commercialization of dark fermentative hydrogen production (Sivagurunathan et al., 2016).

To address instability in continuous dark fermentation, voltage application has been proposed as a strategy to accelerate process stabilization (Akram et al., 2025; Schievano et al., 2016). However, conventional electro-fermentation systems are heavily reliant on surface reactions at the electrode interface, which limits effective reaction sites (Bhagchandanii et al., 2020; Shastri et al., 2025). Dynamic membrane bioreactors (DMBRs) provide a configuration in which a biomass-derived cake layer forms on the membrane surface and acts as a filtration barrier (Saleem et al., 2018). In this structure, the dynamic membrane itself can serve as a biomass-rich interface. Therefore, integrating electrode function into the dynamic membrane module may provide abundant reaction sites in direct proximity to concentrated biomass and facilitate more effective microbe-electrode interaction during high-rate continuous operation.

This study aimed to develop and evaluate an electro-supported dynamic membrane bioreactor (EDMBR) for continuous hydrogen production. The effects of voltage application on stabilization time, hydrogen production performance, metabolic pathway shift, and microbial community change were investigated under different operational conditions. In addition, suspended biomass and biofilm concentrations were analyzed to identify an operational window for rapid stabilization and stable continuous hydrogen production.

## 2 Materials and methods

### 2.1 Reactor configuration

**Figure 1:**
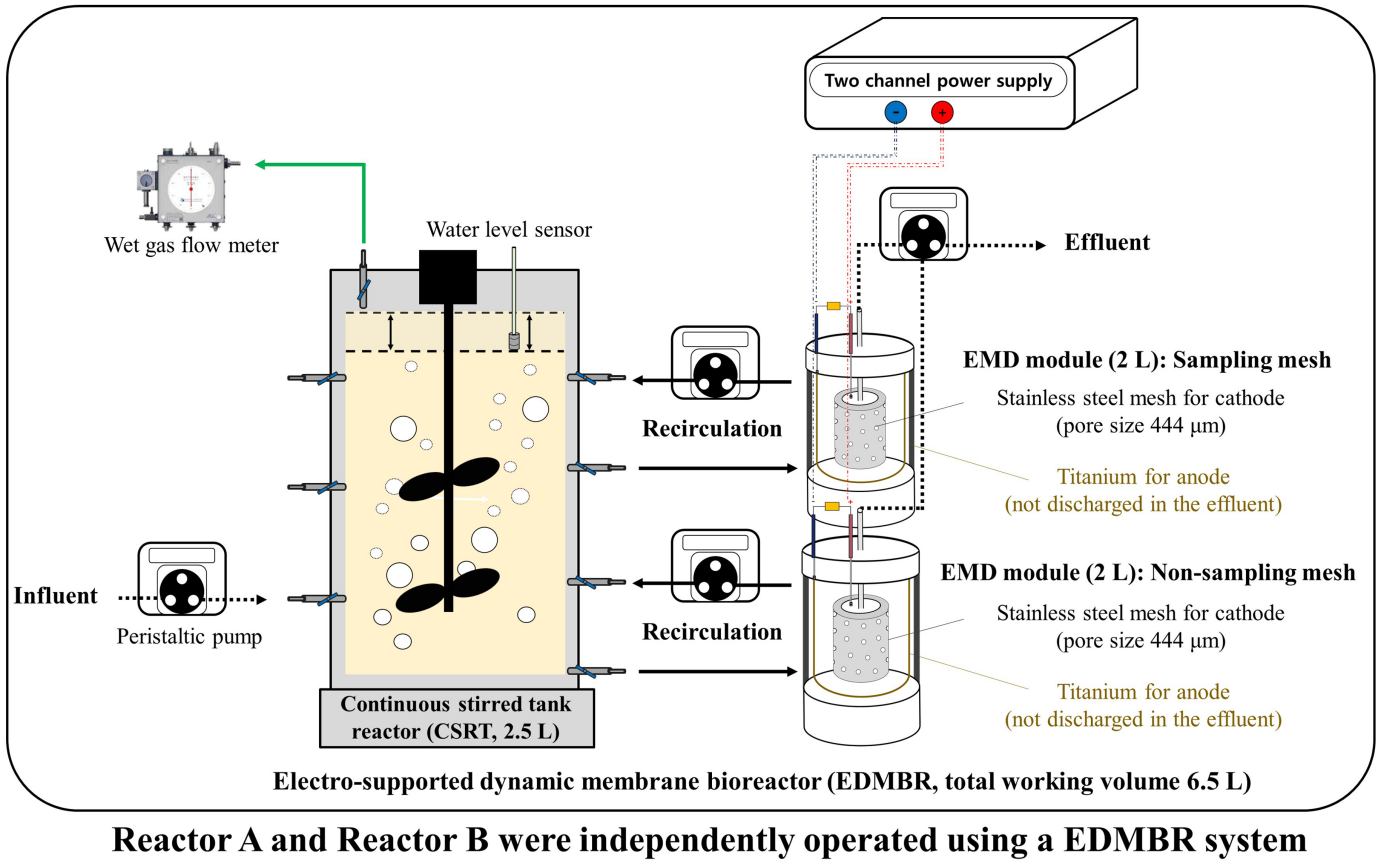
Configuration of the electro-supported dynamic membrane bioreactor (EDMBR) system for continuous hydrogen production.

Two identical lab-scale electro-supported dynamic membrane bioreactors (EDMBR) were constructed by integrating a continuous stirred tank reactor (CSTR) with two side-stream electro-supported dynamic membrane (EDM) modules, and a schematic diagram is presented in Figure 1. Each EDMBR consisted of a single CSTR connected to two side-stream EDM modules, and Reactor A and Reactor B represent two independent EDMBR systems operated under different conditions. The EDM module (393 cm^2^) was equipped with a stainless steel mesh support with a pore size of 444 μm to minimize inoculum and microbial washout, and to allow voltage application on the mesh surface during continuous operation. The stainless steel mesh served as the cathode in this study. A titanium plate was installed at a distance of 100 mm from the cathode to facilitate electron transfer and was used as the anode without additional mesh support. The working volumes of the CSTR and each EDM module were 2.5 L and 2.0 L, respectively, resulting in a total working volume of 6.5 L for the EDMBR system. The working volume was automatically maintained at 6.5 ± 0.1 L (headspace 1.5 ± 0.1 L) using a water-level sensor installed in the CSTR, which controlled the effluent pump in on/off mode. The effluent was discharged through the cathodic EDM modules, allowing permeation across the mesh support, while the influent was continuously supplied to the CSTR using a peristaltic pump. The CSTR served as the main fermentation chamber, where feedstock was continuously supplied and complete mixing was maintained by mechanical stirring at 180 rpm. The EDM modules functioned as filtration units and electroactive interfaces where the dynamic membrane was formed. Mixed liquor was recirculated through the side-stream EDM modules at a shear velocity of 6.75 m/h (corresponding to a recirculation flow rate of 262.0 L/h at an HRT of 2 h), which was determined based on the recirculation flow between the CSTR and the EDM module, thereby facilitating continuous contact between suspended biomass and the electrode surface, and ensuring homogeneous conditions throughout the EDMBR system (Sim et al., 2020). The shear velocity in the EDM modules was defined according to Equation 1.

$$\text{Shear velocity}\;(m/h)=Q_{f}\times \frac{1+R}{A}$$ (1)

Where, *Q_f_* (*m*^3^/*h*) was the feed flow rate, *R* was the ratio between the recirculation flow between CSTR and the DM module and the feed flow rate, and *A* (*m*^2^) was the cross-sectional area of the DM module.

### 2.2 Preparation of inoculum and feedstock

Methanogenic anaerobic sludge was obtained from an anaerobic digester at a wastewater treatment plant in Bucheon, South Korea. The sludge was used as the inoculum and had 18.9 g TS/L, 13.8 g VS/L, and a pH of 6.8. To suppress methanogenic activity, the sludge was thermally pretreated in a water bath at 90 °C for 1 h and subsequently cooled at room temperature for 30 min prior to use. Feedstock was prepared by dissolving glucose at 20 g/L (extra pure 98%, Duksan Pure Chemicals, Korea) in distilled water. The following nutrients were added: NH_4_HCO_3_ (3 g/L), KH_2_PO_4_ (125 mg/L), MgCl_2_·6H_2_O (100 mg/L), FeSO_4_·7H_2_O (25 mg/L), MnSO_4_·6H_2_O (15 mg/L), CuSO_4_·5H_2_O (5 mg/L), and CoCl_2_·5H_2_O (1 mg/L). All chemicals were of analytical grade (≥ 98% purity) and purchased from Duksan Pure Chemicals, South Korea, unless otherwise stated. Sodium bicarbonate (5–10 g/L) was used as a buffering agent, and its concentration was adjusted to maintain reactor pH within the range of 5.5–6.0. Prepared feedstock was stored at 4 °C prior to continuous feeding to minimize substrate degradation.

### 2.3 Continuous operation

The EDMBR was initially filled with 10% (v/v) inoculum and feedstock, and nitrogen gas (N_2_, 99.999%) was then purged into the EDMBR system for 5 min to establish anaerobic conditions. The system was initially operated in batch mode to activate and enrich hydrogen-producing bacteria prior to continuous operation, and was maintained until a hydrogen yield (HY) of 0.5 mol H_2_/mol glucose_added_ was achieved to ensure stable growth of H_2_-producing bacteria (Kim and Shin, 2008). Subsequently, the system was switched to continuous mode with stepwise reduction of hydraulic retention time (HRT) to achieve high-rate hydrogen production. Subsequently, the reactor was switched to continuous mode by supplying feedstock, and hydrogen production rate (HPR) and HY were monitored daily. The hydraulic retention time (HRT) was initially maintained at 6 and 3 h for 4 days each, during which HY was maintained at approximately 1.0 mol H_2_/mol glucose_added_, and was then stepwise reduced as follows previous study (Park et al., 2019). Voltage application was evaluated at an HRT of 2 h, as high-rate continuous hydrogen production rate (>50 L/L/d) can be achieved under this condition (Park et al., 2019). In this study, pseudo-steady-state was defined as periods in which the daily HPR and HY varied within ±10% for at least consecutive 5 days, and each pseudo-steady-state was defined as “Period,” and each operational condition was maintained until reached pseudo-steady-state. The pH was controlled between 5.5 and 6.0 using bicarbonate buffer, and the reactor temperature was maintained at 37 ± 1 °C using a water bath. Samples were collected daily from the middle section of the CSTR and from the EDM module and were immediately stored at −20 °C prior to analysis. One EDM module was removed at the end of each operational condition to assess biofilm amount and microbial community, while the other module remained connected to the CSTR for continuous operation. After sampling, a new screen mesh was installed in the removed EDM module, which was then purged with N_2_ for 3 min and reconnected to the CSTR.

### 2.4 Hydrogen production performance assay

Biogas production was monitored daily using a wet-type gas flow meter (Shinagawa W-NK-0.5BE, Japan) installed at the outlet of the CSTR. Gas samples were withdrawn from the EDMBR headspace with a 150-μL gas-tight syringe and analyzed for hydrogen content using a gas chromatograph (SRI 310, SRI Instruments, and Torrance, CA, United States) equipped with a thermal conductivity detector (TCD) and a Molecular Sieve 5A packed column. The hydrogen fraction was quantified based on calibration with ultra-high purity hydrogen gas. The HPR was determined from the measured cumulative biogas volume and corresponding hydrogen fraction, and the gas volume was normalized to standard temperature and pressure (273.15 K, 1 atm) using the ideal gas equation (Equation 2).

$$\mathrm{HPR}\;(L/L/d)=\frac{\text{Biogas volume}\;(L/d)*\frac{H_{2}\;(\%)}{100}*(\;\frac{273.15}{273.15+37})}{\text{EDMBR working volume}\;(L)}$$ (2)

Effluent collected from the EDM module was filtered through a 0.45 μm membrane prior to chemical analysis. Volatile fatty acids (VFAs) and residual glucose were quantified using high-performance liquid chromatography (HPLC; Waters 717, United States). VFA analysis was performed with an Aminex HPX-87H column (Bio-Rad Laboratories, United States) operated at a flow rate of 0.6 mL/min, employing 5 mM H_2_SO_4_ as the eluent. Residual glucose was determined using a refractive index detector (Waters 410, United States) with ultra-pure tertiary distilled water as the mobile phase at 0.6 mL/min. HYs based on glucose_added_ (HY_added_) and glucose_consumed_ (HY_consumed_) were calculated from the HPR and the corresponding glucose loading rate according to Equation 3. Prior to yield calculation, HPR was converted to “mol H_2_/L/d” using the ideal gas equation.

$$\mathrm{HY}\;(\mathrm{mol}\;H_{2}/\mathrm{mol}\;\text{glucose})=\frac{\mathrm{HPR}(\mathrm{mol}\;H_{2}/L/d)\;}{\frac{\text{glucose concentration}}{\mathrm{HRT}}(\mathrm{mol}\;\text{glucose}/L/d)\;}$$ (3)

### 2.5 Analytical methods

Biofilm samples collected from the EDMBR were subjected to genomic DNA extraction using the PowerSoil DNA Isolation Kit (MoBio Laboratories, United States) following the manufacturer’s instructions. The bacterial 16S rRNA gene was amplified via polymerase chain reaction (PCR) with universal primer sets. The PCR products were subsequently purified to eliminate residual primers and reaction components. Illumina sequencing adapters and sample-specific barcodes were ligated to the purified amplicons to construct sequencing libraries, which were then pooled prior to sequencing. The combined library was analyzed using an Illumina iSeq platform (Illumina, San Diego, CA, United States) according to the standard protocol. Sequence data were processed using the QIIME2 bioinformatics pipeline (v2019.7) (Maki et al., 2023). Taxonomic classification was performed at multiple hierarchical levels, and taxa representing less than 3% relative abundance were consolidated into an “others” category for clarity in visualization and interpretation (Nguyen et al., 2016). Microbial community composition was evaluated at the end of each operational period. Volatile suspended solids (VSS) and chemical oxygen demand (COD) were determined from daily collected samples in accordance with Standard Methods for the Examination of Water and Wastewater (Baird et al., 2017). All physicochemical analyses (except microbial community sequencing) were conducted in triplicate, and results are presented as mean ± standard deviation.

## 3 Results and discussion

### 3.1 Effect of voltage application on continuous hydrogen production performance

Identical two continuous electro-supported dynamic membrane bioreactors (EDMBRs) were operated to minimize the influence of microbial community changes associated with long-term operation and to independently verify the effect of voltage application. Reactor A and B were monitored for 72 days and 59 days, respectively, and the operational conditions were summarized in Table 1.

**Table 1:** Operational conditions of Reactors A and B during continuous electro-supported dynamic membrane bioreactor (EDMBR) operation.

Reactor	HRT (h)	Period	Voltage (mV)	Data collection (d)
Reactor A	2	I	0	20–24
		II	30	33–37
		III	60	46–50
		IV	90	58–62
		V	120	68–72
Reactor B	2	I	0	16–20
		II	150	25–29
		III	300	33–37
		IV	500	42–46
		V	700	50–54

**Figure 2:**
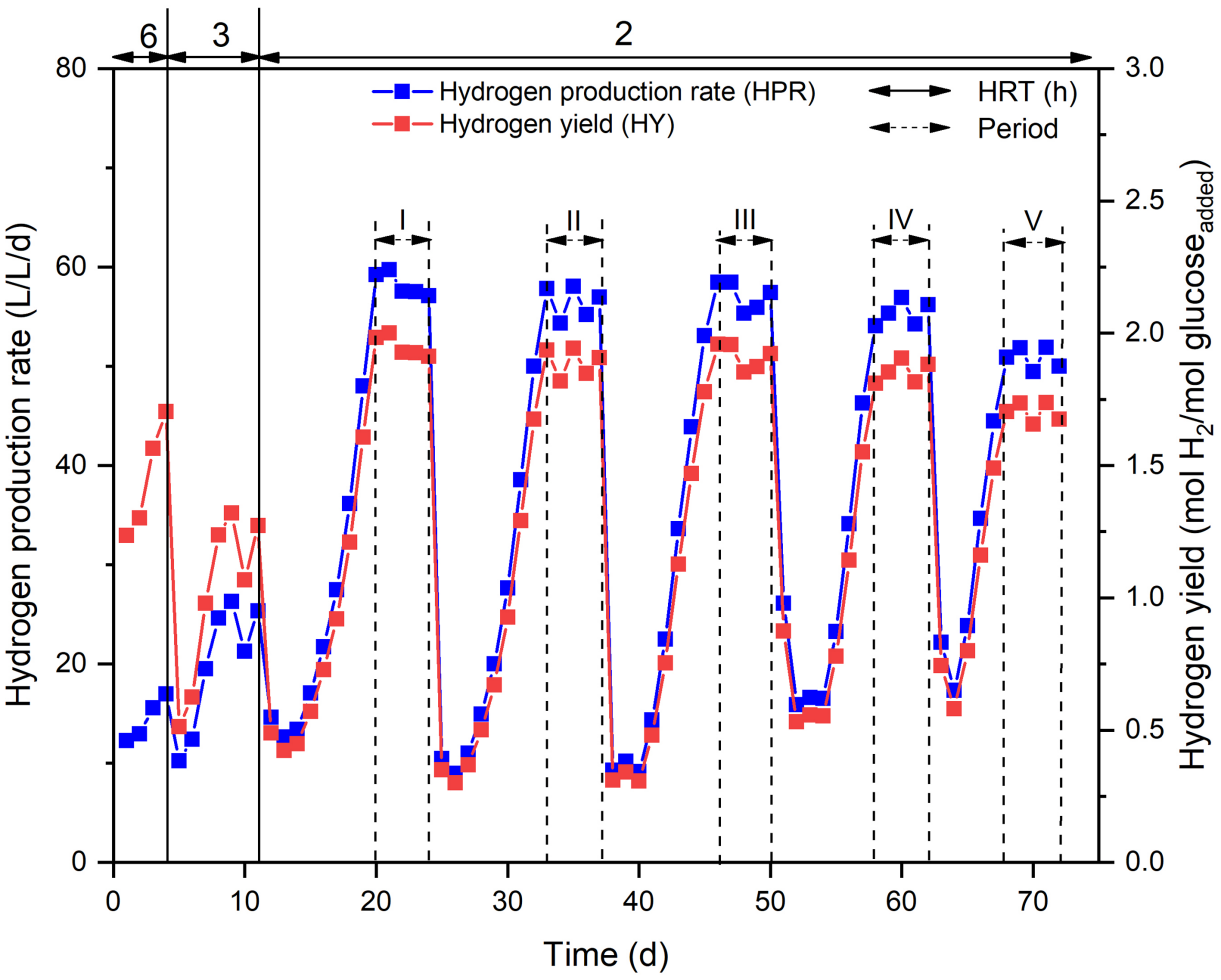
Daily hydrogen production rate (HPR) and hydrogen yield (HY) in control period and voltage application from 30 to 120 mV.

Reactor A was initially operated at HRTs of 6 and 3 h, and the HRT was subsequently switched to 2 h when the hydrogen production rate (HPR) and hydrogen yield (HY) reached 25.35 L/L/d and 1.27 mol H_2_/mol glucose_added_, respectively (Figure 2). Granule formation was observed on day 18, and the reactor reached pseudo-steady-state during days 20–24. This phase was defined as Period I, and exhibited the highest hydrogen production performance with average HPR and HY of 58.22 ± 1.17 L/L/d and 1.95 ± 0.04 mol H_2_/mol glucose_added_, respectively. On day 24, the electro-supported mesh in the EDM module was replaced, and an applied voltage of 30 mV was introduced. During days 33–37 (Period II), the average HPR and HY were recorded as 56.47 ± 1.64 L/L/d and 1.89 ± 0.05 mol H_2_/mol glucose_added_, respectively. In Period III (60 mV, days 46–50), the average HPR and HY were 57.12 ± 1.43 L/L/d and 1.91 ± 0.05 mol H_2_/mol glucose_added_, respectively. In Period IV (90 mV, days 58–62), the average HPR and HY decreased slightly to 55.35 ± 1.22 L/L/d and 1.85 ± 0.04 mol H_2_/mol glucose_added_, respectively. When the voltage was further increased to 120 mV (Period V, days 68–72), the average HPR and HY significantly reduced to 50.82 ± 1.08 L/L/d and 1.70 ± 0.04 mol H_2_/mol glucose_added_, respectively.

**Figure 3:**
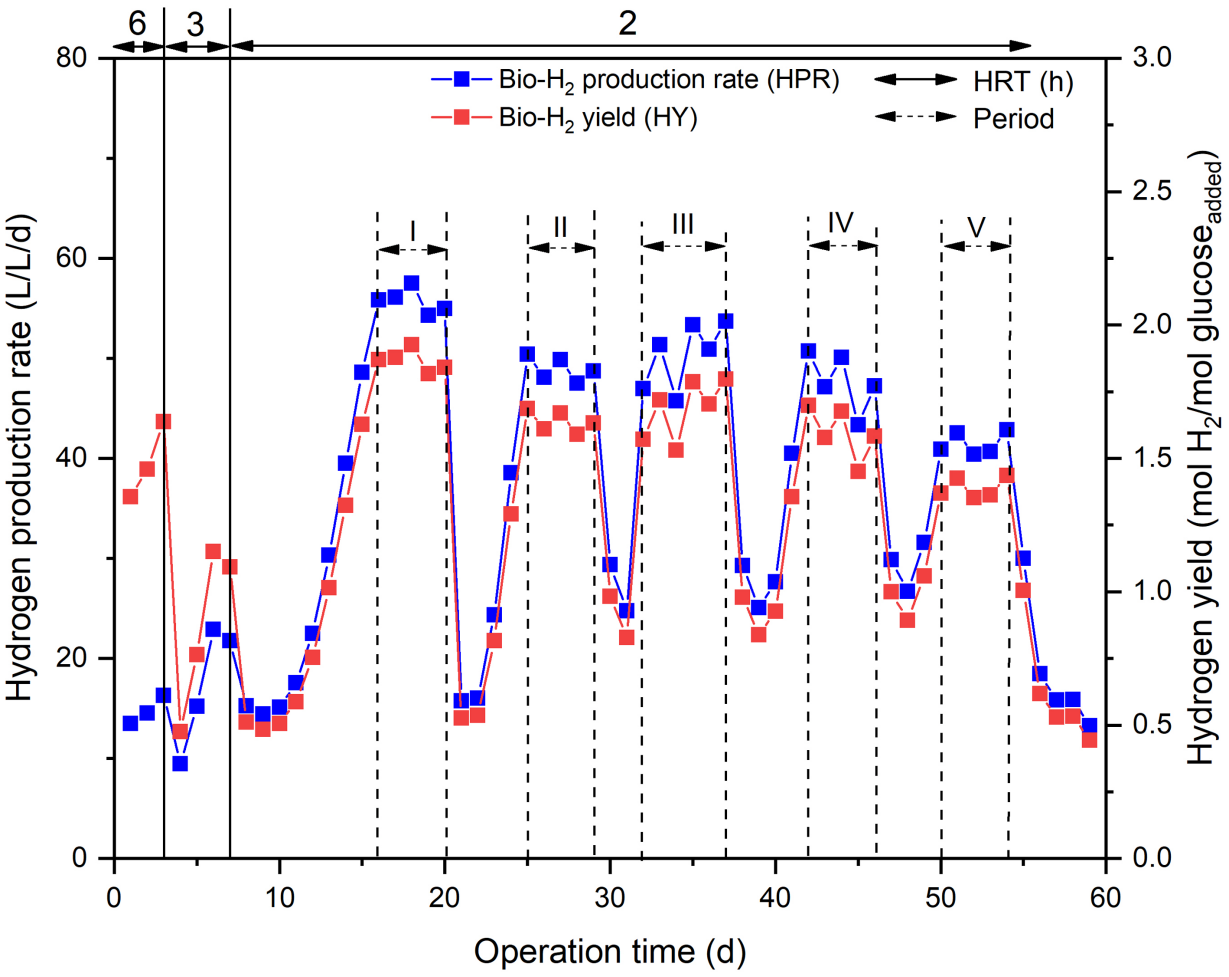
Daily hydrogen production rate (HPR) and hydrogen yield (HY) in control period and voltage application from 150 to 700 mV.

Reactor B was initially operated at HRTs of 6 and 3 h, and the HRT was subsequently switched to 2 h when the HPR and HY reached 21.75 L/L/d and 1.09 mol H_2_/mol glucose_added_ (Figure 3). Granule formation was observed on day 14, followed by an improvement in hydrogen production performance. During Period I (days 16–20), the HPR and HY reached pseudo-steady-state, recording average HPR and HY of 55.74 ± 1.22 L/L/d and 1.87 ± 0.04 mol H_2_/mol glucose_added_, respectively. On day 20, the electro-supported mesh was replaced and a voltage of 150 mV was applied to the EDM module. During Period II (days 25–29), the average HPR and HY were significantly decreased to 48.92 ± 1.20 L/L/d and 1.64 ± 0.04 mol H_2_/mol glucose_added_, respectively. Similar declines were also observed in Period III (300 mV), with average HPR and HY of 51.00 L/L/d and 1.71 ± 0.11 mol H_2_/mol glucose_added_, respectively. This decline suggests that higher voltage application might induce unfavorable metabolic shifts (Moscoviz et al., 2016) and/or progressive process instability during long-term continuous operation (Dahiya et al., 2021). Although the hydrogen production performance did not fully recover to the control condition (Period I), the reactor reached pseudo-steady-state within 4 days after mesh replacement. A similarly rapid stabilization was observed during Periods IV (500 mV) and V (700 mV), but hydrogen production performance was gradually deteriorated with increasing the voltage. Especially, during Period V, the HPR and HY were significantly decreased to 47.71 ± 5.08 L/L/d and 1.60 ± 0.17 mol H_2_/mol glucose_added_, respectively. The reactor performance did not recover, therefore operation was terminated on day 59. Notably, localized corrosion was identified on the mesh during mesh replacement, which might have contributed to reactor deterioration via granule washout and impaired EDM functionality (Ghanim et al., 2025; Kappes, 2020; Li et al., 2023).

Table 2 summarizes the average HPR, HY, stabilization time of Reactors A and B under different operational conditions. In this study, the stabilization time to reach pseudo-steady-state was shortened from the voltage application. At applied voltages of 30–90 mV, 7–8 days were required to establish pseudo-steady-state conditions. When the voltage was increased to 120 mV, the stabilization time decreased to 5 days. Further increases in voltage from 300 to 700 mV shortened the stabilization time to 3–4 days. These results indicate that increasing voltage application facilitates rapid stabilization of continuous EDMBR operation (Guo et al., 2025; Virdis et al., 2022a,b). Although voltage application shortened the time required to reach pseudo-steady-state following mesh replacement, hydrogen production performance in both Reactors A and B was lower than that observed under the control condition. This decline suggests that voltage application may have been associated with shifts in metabolic pathways (Liao et al., 2025; Virdis et al., 2022a,b). In this study, voltage application at 700 mV was associated with rapid stabilization, but it may induce localized corrosion of the electro-supported mesh, thereby compromising stable long-term continuous operation. In this context, when rapid re-establishment of pseudo-steady-state is required following reactor deterioration, voltage application within the range of 120–500 mV for 3–5 days is recommended.

**Table 2:** Average hydrogen production rate (HPR), hydrogen yield (HY), and stabilization time under different voltage applications in Reactors A and B.

Reactor	Period	Voltage (mV)	Hydrogen production rate (L/L/d)	Hydrogen yield (mol H_2_/mol glucose_consumed_)	Stabilization time (d)
Reactor A	I	0	58.22 ± 1.17	1.95 ± 0.04	-
	II	30	56.47 ± 1.64	1.92 ± 0.07	8
	III	60	57.12 ± 1.43	1.91 ± 0.05	8
	IV	90	55.35 ± 1.22	1.88 ± 0.04	7
	V	120	50.82 ± 1.08	1.70 ± 0.04	5
Reactor B	I	0	55.74 ± 1.22	1.91 ± 0.05	-
	II	150	48.92 ± 1.20	1.64 ± 0.04	4
	III	300	51.00 ± 3.19	1.71 ± 0.11	3
	IV	500	47.71 ± 2.92	1.60 ± 0.10	4
	V	700	41.47 ± 1.14	1.60 ± 0.17	3

### 3.2 Distribution of by-products and metabolic flux under voltage application

In dark fermentative bio-H_2_ production, carbohydrate is converted to H_2_, CO_2_, soluble by-products, and microbial biomass, while some of the substrate may remain depending on operational conditions, substrate type, and influent concentration (Jain et al., 2024). However, this conversion can be difficult to track in continuous operation due to dilution and biomass washout via in/effluent, some organic products may not be fully captured within the analytical scope (Kumara Behera and Varma, 2017). In this study, the COD-based mass balance was therefore summarized by accounting for soluble by-products in the effluent, remained hexose, and produced H_2_, and the unaccounted fraction was defined as “biomass and others”. Table 3 summarizes the distribution of soluble by-products in the effluent, residual glucose, hydrogen production, and the overall COD mass balance for Reactors A and B under different operational conditions. In both reactors, butyrate (HBu) was the most abundant soluble metabolite, exceeding 8.14 ± 0.35 g COD/L throughout all operating periods, which is consistent with the typical metabolic characteristics of glucose-based dark fermentation (Gupta et al., 2024). Lactate (HLa) was the second most abundant by-product and increased under voltage-applied conditions. Acetate (HAc) was also affected by voltage application and was identified as the third most abundant soluble metabolite. Although acetate production is generally associated with H_2_-producing pathways in dark fermentation (Albuquerque et al., 2024), HAc concentration exhibited an inverse relationship with hydrogen production performance under voltage-applied conditions. This observation suggests that part of the acetate formation may have been associated with H_2_-consuming pathways, such as homoacetogenesis, rather than exclusively with H_2_-producing metabolism (Saady, 2013). Formate (HFo) and propionate (HPr) were detected at negligible concentrations, remaining below 0.05 ± 0.02 and 0.24 ± 0.06 g COD/L, respectively, throughout the entire operation. This results indicates that butyrate fermentation was the dominant pathway, while the increased fractions of lactate and acetate under voltage-applied conditions suggest a partial shift toward non-H_2_-producing and H_2_-consuming pathways.

**Table 3:** Chemical oxygen demand (COD) distribution of soluble by-products and residual glucose in effluent, hydrogen production during different operational periods in Reactor A and B.

Reactor	Period	Soluble by-products in effluent (g COD/L, A)					Residual glucose (g COD/L, B)	Produced H_2_ (g COD/L, C)	Sum of A, B, C (g COD/L)	Biomass and others (g COD/L)
		HLa	HFo	HAc	HPr	HBu				
Reactor A	I	3.09 ± 0.26	0.03 ± 0.02	2.92 ± 0.18	0.24 ± 0.06	10.80 ± 0.21	0.17 ± 0.08	0.74 ± 0.02	17.99 ± 0.13	3.41 ± 0.13
	II	2.98 ± 0.06	0.03 ± 0.02	2.95 ± 0.16	0.15 ± 0.08	10.81 ± 0.20	0.18 ± 0.07	0.73 ± 0.02	17.84 ± 0.11	3.56 ± 0.11
	III	2.83 ± 0.10	0.02 ± 0.02	2.88 ± 0.29	0.21 ± 0.04	10.91 ± 0.38	0	0.72 ± 0.02	17.57 ± 0.23	3.83 ± 0.23
	IV	3.67 ± 0.15	0.03 ± 0.02	3.38 ± 0.06	0.15 ± 0.08	10.81 ± 0.20	0.02 ± 0.05	0.71 ± 0.01	18.77 ± 0.32	2.63 ± 0.32
	V	4.07 ± 0.15	0.02 ± 0.03	3.36 ± 0.18	0.06 ± 0.08	9.44 ± 0.26	0	0.64 ± 0.01	17.60 ± 0.16	3.80 ± 0.16
Reactor B	I	3.02 ± 0.01	0.05 ± 0.02	2.92 ± 0.18	0.24 ± 0.06	10.77 ± 0.17	0.18 ± 0.08	0.73 ± 0.03	17.92 ± 0.13	3.48 ± 0.13
	II	4.28 ± 0.14	0.04 ± 0.03	3.39 ± 0.04	0.06 ± 0.08	9.27 ± 0.26	0	0.61 ± 0.02	17.67 ± 0.31	3.73 ± 0.31
	III	4.90 ± 0.43	0.03 ± 0.01	3.59 ± 0.31	0.08 ± 0.03	9.22 ± 0.64	0	0.64 ± 0.04	18.45 ± 0.37	2.95 ± 0.37
	IV	6.37 ± 0.27	0.03 ± 0.04	2.72 ± 0.17	0.02 ± 0.03	9.13 ± 0.30	0	0.60 ± 0.04	18.86 ± 0.31	2.54 ± 0.37
	V	6.93 ± 0.33	0.01 ± 0.03	2.56 ± 0.07	0.09 ± 0.10	8.14 ± 0.35	0	0.52 ± 0.01	18.25 ± 0.11	3.07 ± 0.06

In dark fermentative hydrogen production, hydrogen can be theoretically generated at 4 mol H_2_/mol glucose via the HAc-producing pathway (Equation 4) and at 2 mol H_2_/mol glucose via the HBu-producing pathway (Equation 5) (Antonopoulou et al., 2008; Hawkes et al., 2007). In this study, these pathways were categorized as H_2_-producing pathways. However, acetate can be also formed through the H_2_-consuming homoacetogenic pathway (Equation 6), which was therefore classified as an H_2_-consuming pathway. Although HPr and HFo were detected at negligible concentrations, these metabolites are typically associated with H_2_-consuming pathways (Equations 7, 8) and were accordingly grouped within this category (Guo et al., 2010; Nandi and Sengupta, 1998) In contrast, the HLa-producing pathway (Equation 9) does not involve net hydrogen production or consumption (Robergs et al., 2004). However, it competes with H_2_-producing pathways for substrate utilization, and it would therefore influence overall hydrogen production performance (Kenney et al., 2015). So, the HLa-producing pathway was defined as a non-H_2_-producing pathway.

$$C_{6}H_{12}O_{6}+{\mathrm{2H}}_{2}O\to {\mathrm{2C}}_{2}H_{4}O_{2}+{\mathrm{2CO}}_{2}+{\mathrm{4H}}_{2}$$ (4)

$$C_{6}H_{12}O_{6}\to C_{4}H_{8}O_{2}+{\mathrm{2CO}}_{2}+{\mathrm{2H}}_{2}$$ (5)

$${\mathrm{2CO}}_{2}+{\mathrm{4H}}_{2}\to C_{2}H_{4}O_{2}+{\mathrm{2H}}_{2}O$$ (6)

$$C_{6}H_{12}O_{6}+{\mathrm{2H}}_{2}\to {\mathrm{2C}}_{3}H_{6}O_{2}+{\mathrm{2H}}_{2}O$$ (7)

$${\mathrm{CO}}_{2}+H_{2}\to \text{HCOOH}$$ (8)

$$C_{6}H_{12}O_{6}\to {\mathrm{2C}}_{3}H_{6}O_{3}$$ (9)

The theoretical HY was derived from the metabolite balance based on the consumed substrate and the generated by-products (Equation 10), while the difference between the measured and theoretical HY was used to estimate the homoacetogenic flux (Equation 11) (Regueira et al., 2018). Based on this pathway classification and mass balance framework, changes in by-product distribution under different operational conditions were systematically interpreted.

Theoreticalhydrogen yield(molH2/molglucoseconsumed)=2molbutyrate+2molacetate–(1molpropionate+1molformate) (10)

$$\begin{array}{l} \text{Homoacetogenic flux}\;(\mathrm{mol}\;\text{acetate}/\mathrm{mol}\;{\text{glucose}}_{\text{consumed}})\\ =[\begin{array}{l} \text{Theoretical}\;\mathrm{HY}\;(\begin{array}{l} \mathrm{mol}\;H_{2}\\ /\mathrm{mol}\;{\text{glucose}}_{\text{consumed}} \end{array})\\ –\text{Measured}\;\mathrm{HY}\;(\begin{array}{l} \mathrm{mol}\;H_{2}\\ /\mathrm{mol}\;{\text{glucose}}_{\text{consumed}} \end{array}) \end{array}]/4\;\mathrm{mol}\;H_{2}/\mathrm{mol}\;\text{acetate} \end{array}$$ (11)

**Figure 4:**
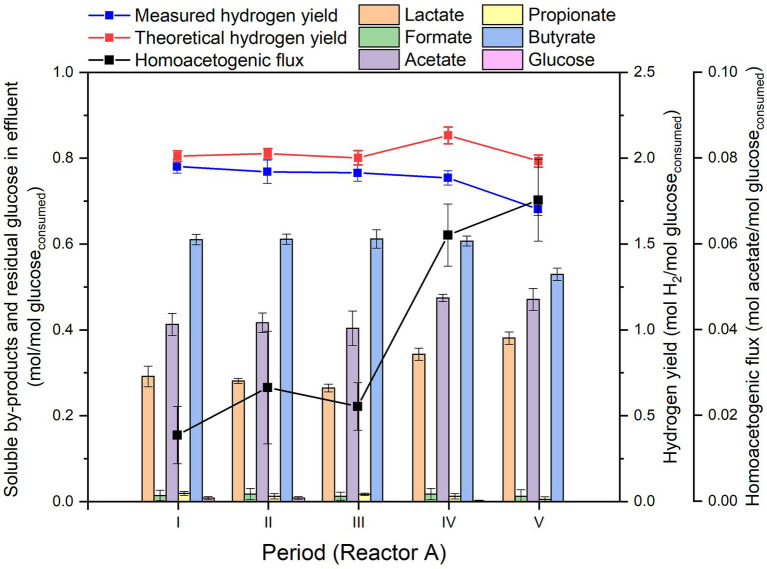
Theoretical measured hydrogen yields (HYs) along with the distribution of soluble by-products and homoacetogenic flux in control period and voltage application from 30 to 120 mV.

Reactor A maintained comparable theoretical and measured HYs, along with a consistent distribution of soluble by-products during Periods I–III (Figure 4). These observations indicate that voltage application at 30 and 60 mV had a limited influence on hydrogen production performance and by-product distribution. More noticeable changes in by-product distribution were observed during Periods IV and V. In Period IV, the measured HY and HBu were similarly maintained with previous voltage conditions, whereas the homoacetogenic flux and HLa were increased to 0.07 ± 0.01 and 0.34 ± 0.01 mol/mol glucose_consumed_, respectively. In Period V, the homoacetogenic flux and HLa were more increased to 0.07 ± 0.01 and 0.38 ± 0.01 mol/mol glucose_consumed_, respectively, beginning the measured HY and HBu were decreased to 1.70 ± 0.04 and 0.53 ± 0.02 mol/mol glucose_consumed_, respectively.

**Figure 5:**
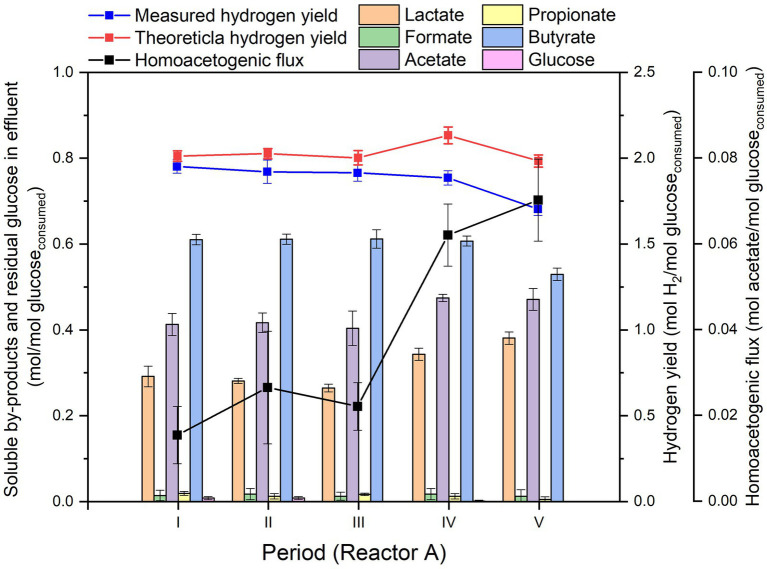
Theoretical measured hydrogen yields (HYs) along with the distribution of soluble by-products and homoacetogenic flux in control period and voltage application from 150 to 700 mV.

Reactors B exhibited similar hydrogen production performance and metabolite distribution with Reactor A during the control period (Figure 5). However, measured HY and HBu were significantly decreased under further voltage-conditions. In Period II, measured HY and HBu were decreased to 1.64 ± 0.04 and 0.52 ± 0.02 mol/mol glucose_consumed_, respectively, while the homoacetogenic flux and HLa increased to 0.08 ± 0.01 and 0.40 ± 0.01 mol/mol glucose_consumed_, respectively. These results suggest that voltage application was associated with a reduced relative contribution of H_2_-producing pathways and an increased contribution of homoacetogenic and HLa-producing pathways. In Periods IV and V, although the homoacetogenic flux was temporarily decreased to 0.04 ± 0.01 and 0.06 ± 0.01 mol acetate/mol glucose_consumed_, respectively, HLa was elevated by voltage application.

In this study, the decline in hydrogen production performance under voltage application may be associated with a redistribution of metabolic pathways from H_2_-producing pathways toward homoacetogenic and lactate-producing pathways. This interpretation is supported by the observed changes in metabolite distribution, where the relative contribution of butyrate decreased while lactate and acetate concentrations increased under voltage-applied conditions. In particular, the increase in reduced metabolites such as lactate, along with the decrease in hydrogen production performance, suggests that a portion of the reducing equivalents was redirected from H_2_-producing pathways toward soluble metabolite formation. Considering the relative changes in hydrogen yield and metabolite profiles, this trend indicates an approximate redistribution of electron flow within the system, where electrons were partially consumed in competing pathways rather than being converted into hydrogen. However, this performance decline may not be solely attributed to metabolic shifts, but may also be influenced by changes in biomass retention and microbial community. Therefore, the following section examines variations in biofilm formation and suspended biomass concentration under different operational conditions, with a primary focus on biofilm in relation to microbial community dynamics.

### 3.3 Impact of voltage application on microbial community

Table 4 summarizes the current density, biofilm concentration, and suspended biomass concentrations in the reactor and effluent for Reactors A and B under different operational conditions. As shown in Table 4, as the applied voltage increased, the measured current and corresponding current density also increased, reflecting a higher electrochemical driving force under each condition. A notable common feature between Reactor A and B (except for Reactor B in Period V) was that biofilm concentration remained relatively stable during the entire operation, ranging from 52.19 ± 0.97 to 75.48 ± 0.39 mg VSS/cm^2^ under voltage application. Considering the shortened time required to reach pseudo-steady-state under voltage application, it is reasonable to suggest that electrical input promoted the biofilm formation. In addition, suspended biomass in the reactor generally increased under voltage application, while suspended biomass in the effluent decreased, indicating enhanced biomass retention. It imply that voltage application have contributed to promotion of biofilm formation and the maintenance of higher biomass concentrations within the reactor. For example, Liao et al. (2025) reported that electrode biofilm thickness increased under voltage application at 0.6 V, while Pous et al. (2023) demonstrated that biomass concentration and growth rate were enhanced under voltage application. In this context, voltage application would be associated with promoted biofilm formation, which prevented suspended biomass washout, thereby facilitating the maintenance of higher suspended biomass concentrations within the reactor. In contrast, localized corrosion of the mesh surface was observed in Reactor B during Period V, so the concentration of biofilm and suspended biomass in the reactor were markedly decreased to 33.42 ± 0.19 mg VSS/cm^2^ and 6.48 ± 2.40 g VSS/L, respectively. This decline was accompanied by reduced hydrogen production performance. Therefore, this study recommends that the current density below 1.62 ± 0.78 mA/cm^2^ to avoid the localized corrosion for stable EDMBR operation.

**Table 4:** Current density, suspended biomass concentration, and biofilm concentration under different voltage applications in Reactors A and B.

Reactor	Period	Voltage (mV)	Current density (mA/cm^2^)	Suspended biomass in the reactor (g VSS/L)	Suspended biomass in the effluent (g VSS/L)	Biofilm concentration (mg VSS/cm^2^)
Reactor A	I	0	0	7.49 ± 0.07	2.48 ± 0.06	53.94 ± 1.01
	II	30	0.07 ± 0.01	7.45 ± 0.01	2.47 ± 0.01	52.19 ± 0.97
	III	60	0.15 ± 0.01	7.88 ± 0.08	2.42 ± 0.03	53.01 ± 0.77
	IV	90	0.23 ± 0.02	8.06 ± 0.03	2.25 ± 0.04	53.01 ± 0.77
	V	120	0.30 ± 0.01	8.73 ± 0.35	2.12 ± 0.06	55.41 ± 0.68
Reactor B	I	0	0	7.32 ± 0.11	2.67 ± 0.27	52.49 ± 1.39
	II	150	0.37 ± 0.02	8.96 ± 0.06	2.09 ± 0.04	55.41 ± 0.10
	III	300	0.75 ± 0.02	10.37 ± 0.33	1.98 ± 0.03	55.48 ± 0.39
	IV	500	1.21 ± 0.09	9.14 ± 0.06	2.14 ± 0.03	52.88 ± 0.97
	V	700	1.62 ± 0.78	6.48 ± 2.40	4.42 ± 2.12	33.42 ± 0.19

**Figure 6:**
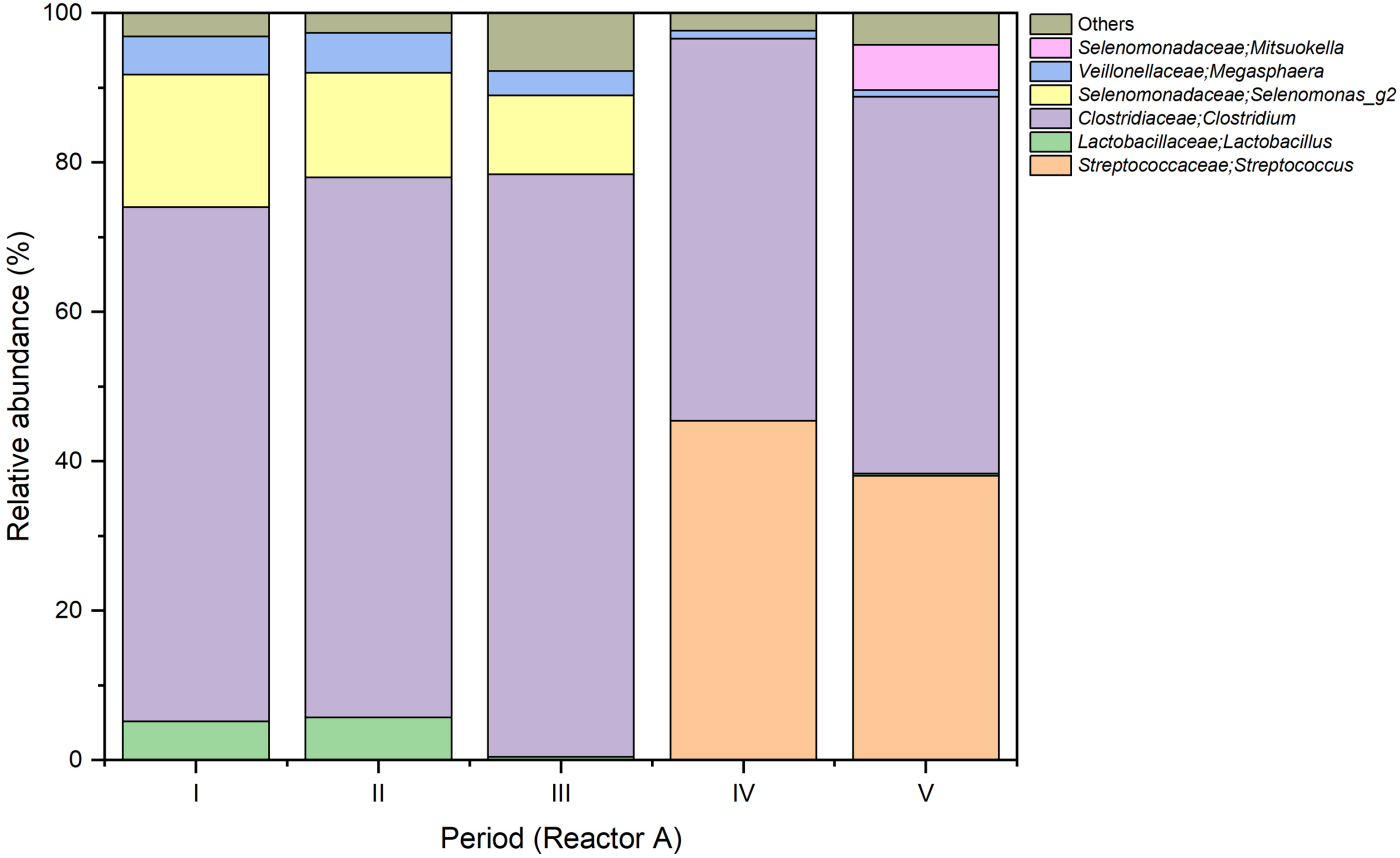
Relative abundance of microbial community at species level in control period and voltage application from 30 to 120 mV.

Reactor A exhibited substantial changes in microbial community with increasing voltage application (Figure 6). Throughout the entire operation, the H_2_-producing bacteria, *Clostridium* were the dominant genus. During Period I, relative abundance of *Clostridium* was 68.80% in the total bacteria, and it was the most abundant genus, but its relative abundance was markedly decreased to 50.45% in Period V. Recent studies have been reported that *Clostridium* sp. can contributes to hydrogen production as electroactive microorganisms by directly interacting with cathodic surfaces (Zani et al., 2024; Zhang et al., 2023), and this microbiome trend is consistent with previous studies in electro-fermentation (Arbter et al., 2022; Moscoviz et al., 2017; Toledo-Alarcón et al., 2019). Meanwhile, the relative abundances of *Streptococcus* was significantly increased along with homoacetogenic flux and HLa concentration in Period IV and V. These bacteria may be associated with homoacetogenic- and HLa-producing pathways (Greening et al., 2019; Park et al., 2023). In addition, *Megasphaera,* *Lactobacillus*, *Selenomonas_g*2 were detected in Reactor A during the early operational period (I-III), but their relative abundances progressively decreased with increasing voltage application, and negligible during Period IV and V, suggesting that elevated voltage conditions might have reduced the relative abundances of these genera.

**Figure 7:**
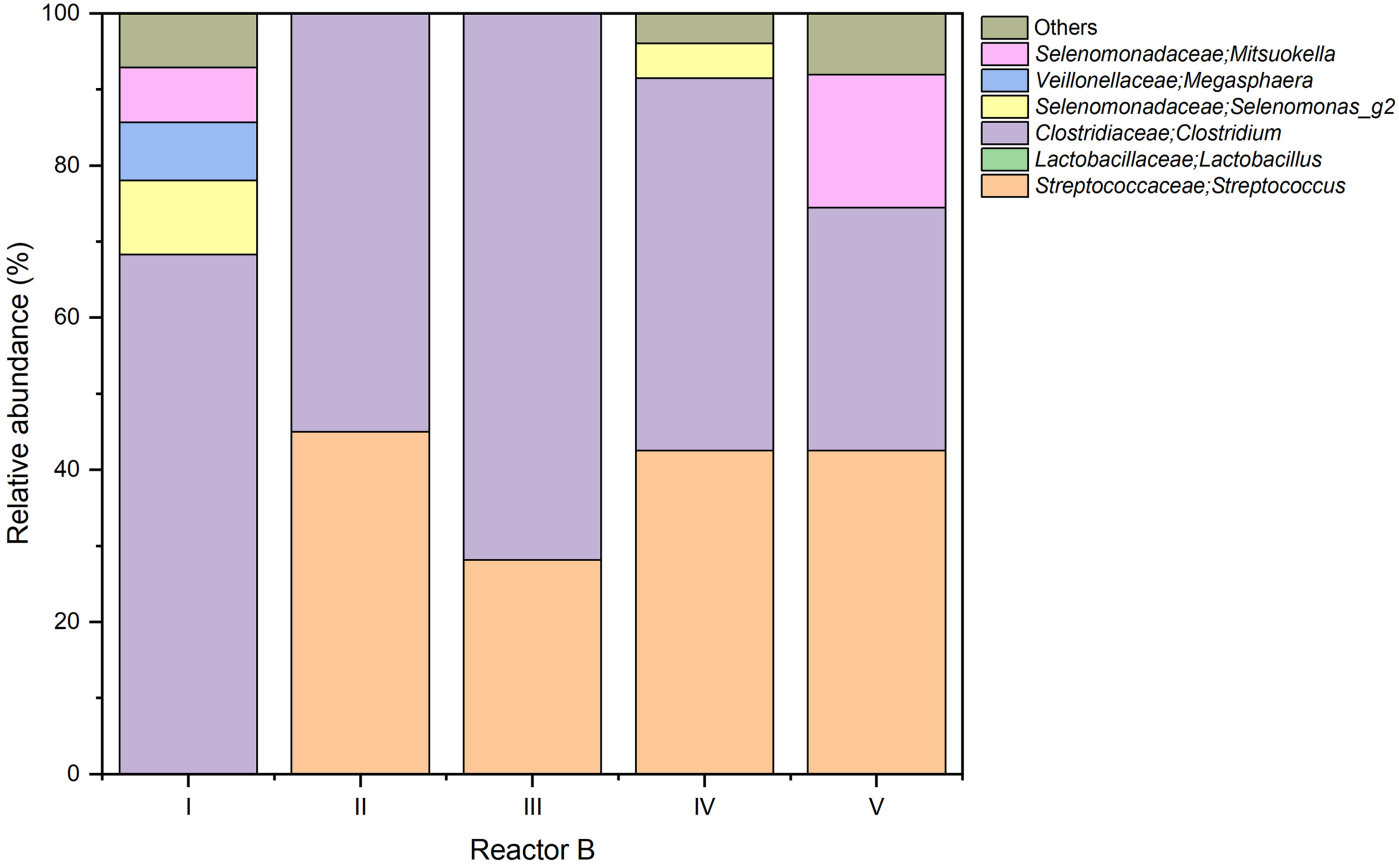
Relative abundance of microbial community at species level in control period and voltage application from 150 to 700 mV.

Reactor B exhibited a microbial community similar to Reactor A during the control period (Period I), but more dynamic shifts were observed under subsequent voltage conditions (Figure 7). In Period I, *Clostridium* was the dominant genus as 68.27% of the total bacteria, and its relative abundance were markedly decreased to 31.91% in Period V. Meanwhile, the relative abundance of *Streptococcus* significantly increased from 0% to 45.0% between Periods I and II, and temporarily decreased to 28.18% in Period III, and subsequently increased again to 42.52% and 42.53% under the subsequent voltage conditions. These microbial community shifts coincided with reduced hydrogen production performance and increased HLa production under elevated voltage conditions. Previous studies have reported that *Streptococcus* *spp*. are frequently observed in electro-fermentation systems, and these genera might contribute to the instability of hydrogen production by outcompeting H_2_-producing bacteria (Chen et al., 2016; Toledo-Alarcón et al., 2019). In particular, *Streptococcus spp*. became the dominant genus in Period V along with reactor deterioration in this study, and this observation is consistent with previous studies in electro-fermentation systems (Chen et al., 2016; Toledo-Alarcón et al., 2019). In addition, no other microbial genera except *Clostridium* and *Streptococcus* were detected during voltage application at 150-300 mV (Periods II and III), indicating that these voltage application might have selectively enriched specific bacterial genera associated with H2-producing and HLa-producing pathways. Consequently, voltage application was associated with a shift in metabolic flux from H2-producing pathways toward homoacetogenic- and HLa-producing pathways, which may have been related to the increased relative abundances of *Streptococcus*. These metabolic and microbial community changes may have contributed to the decline in hydrogen production performance.

Overall, voltage application in the continuous EDMBR system was associated with two contrasting effects on reactor performance. On one hand, electrical input facilitated rapid stabilization, which was related to enhanced biofilm formation and improved biomass retention. On the other hand, sustained voltage application was associated with changes in metabolic flux distribution and microbial community structure during continuous operation. This result clearly indicated a trade-off between rapid stabilization and hydrogen production performance under voltage application. While excessive electrical input may compromise hydrogen production efficiency, controlled voltage application can be beneficial as a tool for rapid stabilization and recovery of reactor performance during continuous operation, particularly under conditions where system instability or performance deterioration occurs. In particular, instability and performance deterioration are major barriers to long-term operation in dark fermentation, and the ability to rapidly restore pseudo-steady-state represents a significant operational advantage. Therefore, voltage application should not be considered as a strategy to continuously enhance hydrogen production performance, but rather as an operational tool to be applied selectively during reactor recovery and/or rapid stabilization.

In the present EDMBR system, the electrode integrated into the dynamic membrane module is considered to influence both electrochemical and biological processes. At the cathode, reduction reactions may have occurred, including proton reduction and hydrogen evolution, as well as possible direct or indirect electron transfer to electroactive microorganisms (Logan et al., 2019; Moscoviz et al., 2016). These processes could alter the intracellular redox balance and consequently influence metabolic pathway distribution during fermentation (Schievano et al., 2016; Virdis et al., 2022a,b). At the anode, oxidation reactions may have contributed to electron withdrawal from the system, thereby affecting overall electron flow and redox conditions. In addition, electrochemical reactions at both electrodes may have influenced local environmental conditions, such as pH and oxidation–reduction potential (ORP), which are known to regulate microbial activity and community structure in electro-fermentation systems (Harnisch and Schröder, 2010; Moscoviz, 2017). Although the exact reaction mechanisms were not directly quantified in this study, these electrochemical interactions provide a plausible explanation for the observed changes in metabolic flux distribution and microbial community under voltage-applied conditions.

In this context, additional operational strategies may be necessary to maintain H_2_-producing pathways while preserving the rapid stabilization benefits of voltage application. Sim et al. (2024) reported that bioaugmentation with *C. pasteurianum* significantly enhanced hydrogen production performance by shifting the microbial community from competing populations toward H_2_-producing bacteria. This strategy may represent a promising approach for EDMBR systems to mitigate performance deterioration by enriching the relative abundance of H_2_-producing bacteria. In addition, intermittent voltage application has been reported to improve hydrogen production performance (Cho et al., 2019). For example, Seo et al. (2024) demonstrated that duty-cycled voltage application (15 s ON/5 s OFF) increased HY by 14.3% in a microbial electrolysis cell system. This strategy may offer a promising approach to enhance hydrogen production performance in the EDMBR by maintaining metabolic flux toward H_2_-producing pathways. Application of these strategies to EDMBR systems may enable both rapid stabilization via voltage application and high-rate continuous hydrogen production. Furthermore, this study evaluated hydrogen production performance in an EDM module where both the cathode and anode were simultaneously submerged. Under this configuration, it would be difficult to accurately distinguish and interpret the individual reaction mechanisms occurring at the cathode and anode based solely on the present dataset, as electrochemical and microbial processes are inherently coupled in bioelectrochemical systems and often occur simultaneously at both electrodes (Harnisch and Schröder, 2010; Logan et al., 2019). In addition, the measured current and voltage responses do not provide direct information on specific reaction pathways, making it challenging to differentiate the contributions of cathodic reduction and anodic oxidation processes (Moscoviz et al., 2016; Schievano et al., 2016). Therefore, further studies are recommended to elucidate the individual reaction mechanisms occurring at each electrode and to optimize the operational voltage and current density ranges based on mechanistic understanding. In particular, additional investigations on electron transfer mechanisms and hydrogen balance are necessary to better understand the redistribution of reducing equivalents under voltage-applied conditions. Such investigations would provide deeper insight into the underlying electrochemical mechanisms and their interactions with the microbial community.

## 4 Conclusion

This study evaluated the effects of voltage application on continuous hydrogen production performance using an electro-supported dynamic membrane bioreactor (EDMBR). Voltage application significantly reduced the stabilization time required to reach pseudo-steady-state from 8 days to 3 days, which was associated with enhanced biofilm formation and improved biomass retention within the reactor. However, voltage application was also associated with a shift in metabolic flux from H_2_-producing pathways toward homoacetogenic- and lactate-producing pathways, along with changes in microbial community, resulting in decreased hydrogen production performance under elevated voltage conditions. These findings highlight a fundamental trade-off between electrochemically enhanced stabilization and biologically driven hydrogen production efficiency in the continuous EDMBR systems. Furthermore, current density at 1.62 ± 0.78 mA/cm^2^ induced localized mesh corrosion, compromising biomass stability and leading to reactor deterioration. An optimal operational window ranging from 0.30 to 1.21 mA/cm^2^ was identified to achieve accelerated stabilization while minimizing the risk of corrosion and severe reactor deterioration. From a practical perspective, voltage application in the continuous EDMBR systems should be strategically managed rather than continuously intensified, because excessive electrical input may accelerate stabilization while simultaneously compromising long-term hydrogen production performance. Further studies are recommended to develop integrated operational strategies, including bioaugmentation and intermittent voltage application, to maintain metabolic flux toward H_2_-producing pathways and preserve the dominance of H_2_-producing bacteria for stable and high-rate continuous hydrogen production using an EDMBR.

## Data Availability

The datasets generated during the current study have been deposited in the NCBI Sequence Read Archive (SRA) under BioProject accession number PRJNA1473093.
